# 

**Published:** 1875-04-15

**Authors:** 


					﻿No. VIII.
APRIL 16, 1876.
Vol. XVI.
THE
Medical Examinee:
A.
wini -11 onthlg :|loui|iral of lledital fcontra.
EDITED BY
N. S. DAVIS, M. D., and F. H. DAVIS, M. D.
Subscription Price, Three Dollars per Annum, in advance
Single .Numbers, Fifteen Cents.
^CHICAGO-
PUBLISHED ArT Go E. RANDOLPH STREET,
				

